# Extremely rare case of intravascular solitary fibrous tumour in the inferior vena cava with review of the literature

**DOI:** 10.1186/s13000-019-0862-9

**Published:** 2019-08-07

**Authors:** Jingyuan Fan, Jinfeng Qiu, Qingjun Wei

**Affiliations:** 1 Department of Orthopedics and Traumatology Surgery, The First Affiliated Hospital of Guangxi Medical University, Nanning, Guangxi 530021 People’s Republic of China; 2 Department of Oncology, The First Affiliated Hospital of Guangxi Medical University, Nanning, Guangxi 530021 People’s Republic of China

**Keywords:** Solitary fibrous tumor, Inferior vena cava, Next generation sequencing

## Abstract

**Background:**

Solitary fibrous tumour (SFT) is a mesenchymal tumour of fibroblastic type, and it develops in almost any part of the human body. However, according to previous studies, the occurrence of intravascular SFTs is extremely rare.

**Case presentation:**

We reported a case of intravascular SFT in a 67-year-old woman who has been experiencing swelling and pain in the right leg for 2 months. Computed tomography venography scan revealed a well-defined mass obstructing the inferior vena cava (IVC). Surgical resection was performed, and histopathologic and immunohistochemical results were consistent with SFT. Further, next-generation sequencing (NGS) analysis was performed, and results revealed two tumour-related gene mutations (deletion of PMS2 and variation of ESR1 [L536P]). The patient did not receive any adjuvant therapy, and no signs of tumour progression were observed during the 6-month follow-up.

**Conclusion:**

To the best of our knowledge, this study first presented about SFT arising from the IVC and carried out an NGS analysis to validate the molecular mechanism of such condition.

## Introduction

Solitary fibrous tumour (SFT) is a mesenchymal tumour of the fibroblastic type, typically arising from the pleura [1]. This type of tumour was first described by Klemperer and Rabin in 1931 [2]. For several years, SFTs were considered as mesothelial tumours arising from the pleura. However, such tumours were recently observed in almost every region of the human body. The tumour is predominantly observed in middle-aged individuals, with no gender difference [3]. Most tumours are benign, but approximately 12–22% [4] of cases exhibit aggressive behaviours, such as local recurrence and metastasis [5]. Different treatment methods for such tumours are reported. However, surgical excision is still considered to be the best option for diagnosis and treatment [6]. Moreover, pathological and immunohistochemical examinations are essential for diagnosis, and the fusion of NAB2-STAT6 was identified as the latest molecular hallmark [7]. Although extrapleural solitary fibrous tumours had been well described in previous studies, the incidence of intravascular SFTs is extremely rare. Herein, we present a case of SFT arising from the inferior vena cava (IVC) in a 67- year-old woman who was preoperatively diagnosed with thrombus in the IVC.

### Case history

**Fig. 1:**
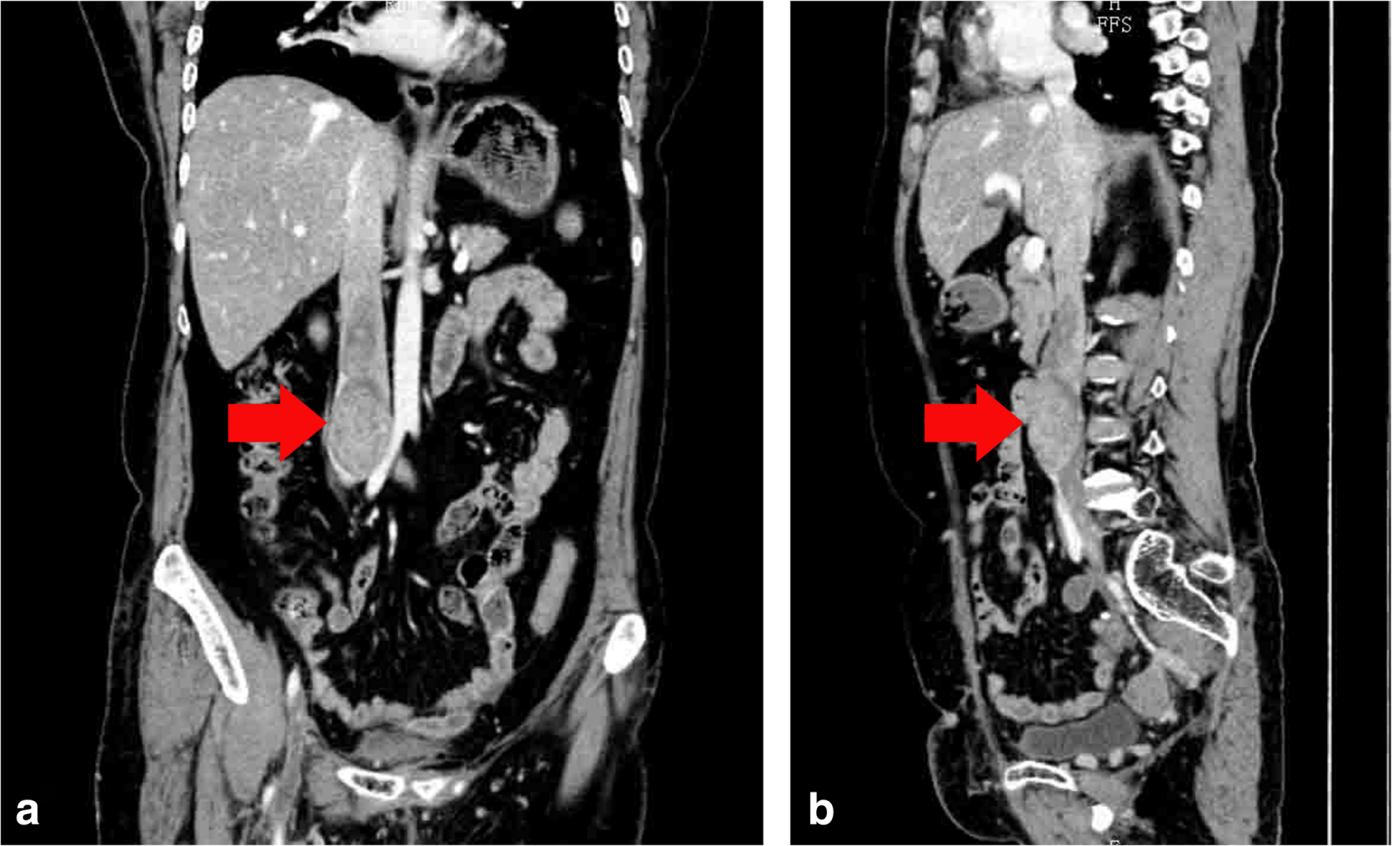
Computed tomography venography characteristics of the neoplasm. **a**. coronal plane; **b**. sagittal plane

A 67-year-old woman was admitted to our hospital. She complained of swelling and pain in the right leg. She claimed that the swelling was not apparent during onset, which was approximately 2 months back, and it subsided over the past days. Moreover, she had developed persistent pain for 7 days before hospitalization. The patient denied any other symptoms, including physical disturbance, weight loss, or fever. She was not a smoker, did not present with hypertension or diabetes and had undergone complete surgical resection of the uterus in 2005. Colour Doppler ultrasonography indicated that the veins in the right lower limb were completely occluded. Computed tomography venography scan of the abdomen and both lower extremities revealed that a well-defined mass obstructed the inferior vena cava (IVC), with segmental filling defect of the right iliac vein and occlusions in the right femoral, popliteal and calf veins (Fig. 1).

For further diagnosis and treatment, surgical excision of the intravascular mass was performed. Under blood blocking, a transverse incision was made in the wall of the inferior vena cava. Next, the 5 × 4 × 3-cm mass with white capsule was cautiously separated from the venous wall. The mass was resected along with part of the venous wall because some tumour tissues were connected to the intravascular neoplasm. Because the tumour adhered tightly to the right common iliac vein and could not be completely resected, a partial resection was carried out. In addition, intraoperative observation revealed that the collateral branch veins from the posterior wall had abundant blood supply, whereas the distal vena cava was completely embolized. Thus, vena cava resection was conducted.

**Fig. 2:**
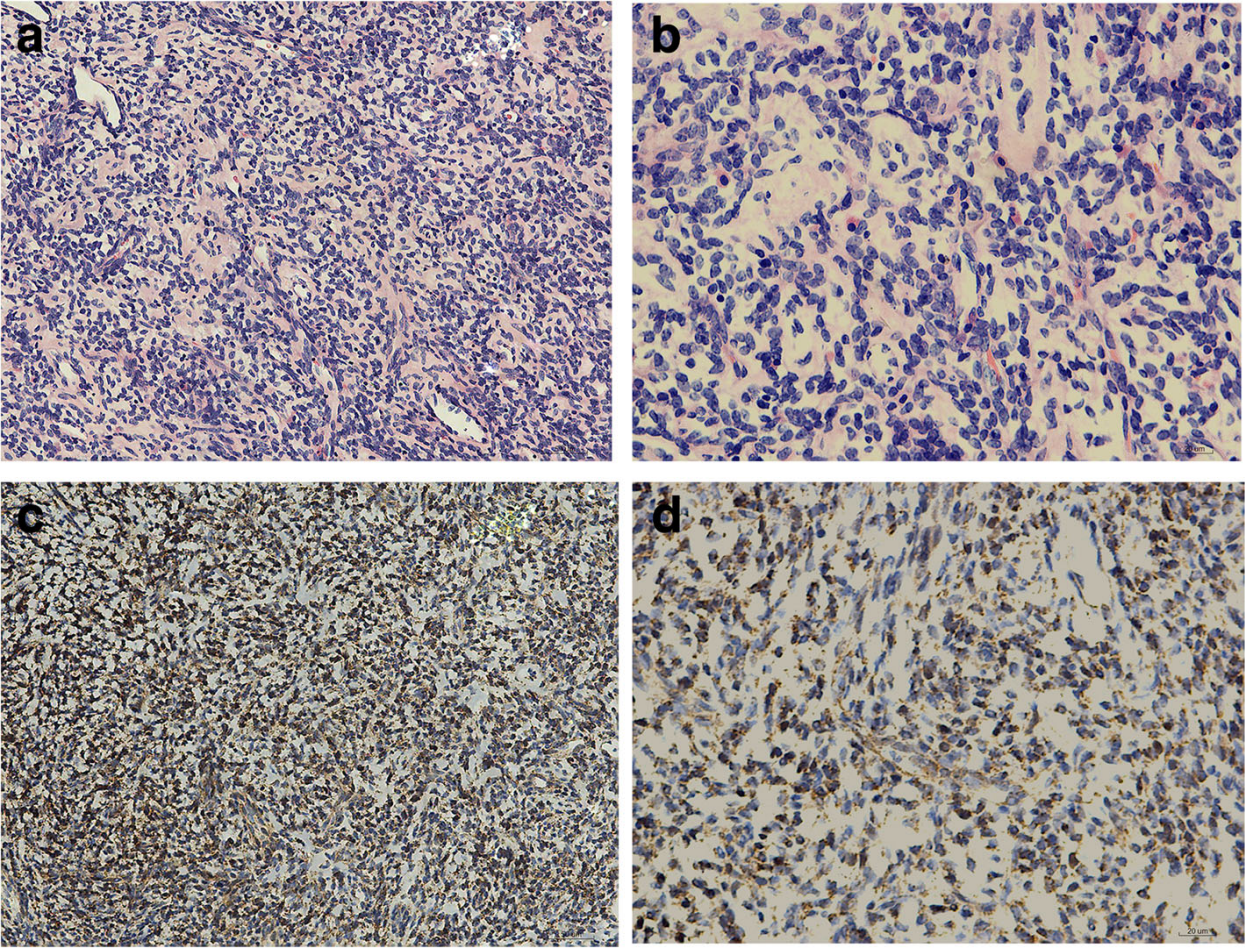
Histological examination and immunohistochemical staining of the neoplasm **a** and **b**: Haematoxylin and eosin staining section showing a hypercellular fibrillary neoplasm. **c** and **d**: Immunohistochemistry with the antibody of STAT6 showing strong nuclear expression

The cut surface was similar to that of other mesenchymal neoplasms, with a fish flesh soft grey-white appearance (Fig. 2a, b). The haematoxylin and eosin staining images of the excised mass revealed that the tumour contained spindle cells. Immunohistochemical analysis showed that the tumour was positive for vimentin, CD99, Bcl-2 and CD34 and negative for D2–40, desmin, S-100, SMA and CK and the Ki-67 index was as low as 1%. In addition, the tumour was positive for STAT6, which is a specific marker of SFT (Fig. 2c, d). According to the imaging characteristics, morphology and immunophenotype, the patient was finally diagnosed with extrapleural SFT.

The patient presented with intestinal obstruction after surgery, which was relieved with pharmacologic and symptomatic treatments for 4 weeks. She was discharged on day 30 after surgery and did not undergo chemotherapy or radiotherapy. During the 6-month follow-up, ultrasonography was conducted, and progression of the disease was not observed.

### Next-generation sequencing

Formalin-fixed paraffin-embedded (FFPE) tissues were manually macrodissected. DNA was extracted from the original tumour, and blood samples were collected using the QIAamp DNA FFPE Tissue Kit (Qiagen). Next-generation sequencing (NGS) was conducted on the MiSeq platform using a TruSeq Amplicon Cancer Panel, covering 520 tumour-related genes. Then, the results were analysed using the MiSeq Reporter software.

NGS results indicated two tumour-related mutations (deletion of PMS2 and variation of ESR1 (L536P)), and the tumour mutation burden was approximately < 0.8 Muts/Mb. In addition, the microsatellite stability was assessed, and the tumour was classified as microsatellite stable.

## Discussion

Solitary fibrous tumour (SFT) is a rare type of neoplasm of mesenchymal origin, mostly arising from the submesothelial connective tissue beneath the pleura [8]. However, the incidence of extrapleural SFT (ESFT) has been increasing within the last two decades, and approximately more than one-third of literature in English is about ESFTs [9]. Such tumour has been observed at almost every extrapleural anatomic site, including the liver [10], kidney [11], pancreas [12], prostate [13], breast [14], central nervous system [15], reproductive system [16, 17], orbit [18], head and neck [19], pelvis [20], soft tissues in the extremities [21] and retroperitoneum [22]. However, the incidence of intravascular SFT is extremely rare [23]. Previous studies have presented cases of SFT invading or involving the IVC [24, 25]. Thus far, this report first presented about SFT arising from the IVC and conducted an NGS analysis.

Like most SFTs, the initial presentation of intravascular SFT depends on tumour size, location, and adjacent anatomic structures. Based on our review, intravascular SFT can be asymptomatic and incidentally diagnosed on imaging examinations, or it can cause venous obstruction at advanced stage. In addition, some patients with SFTs may present with constitutional symptoms, such as hypoglycaemia [26] due to secretion of insulin-like growth factors and weight loss [27], which has not been reported in intravascular cases.

As the symptoms and imaging results are not typical, the preoperative diagnosis of such disease is extremely challenging. In such location, it can be misdiagnosed as phlebothrombosis or other more common tumours, such as leiomyomas, leiomyosarcoma [28], well-differentiated/dedifferentiated liposarcoma [25], intimal sarcoma [29] and haemangiomas [30]. Clinical and imaging features also overlap. Therefore, the diagnosis of intravascular SFT is hardly achieved until surgical excision and pathological examination are carried out [31].

The patient presented with a well circumscribed spindle cell tumour with a complete capsule. The cellular area was hypervascular, with classical patternless pattern of proliferation. Cellularity ranged from hypercellular to laxis areas surrounding varying amounts of hyalinized collagen bundles [5]. Based on immunohistochemical examination, the tumour cells were positive for CD34, Bcl2, vimentin, and CD99 and negative for desmin and S-100 [7]. Zhanlong, M. et al. have presented compelling results showing that immunohistochemical results might be associated with the origin and site of the tumour [32], which is significant in making a differential diagnosis. However, further studies must be conducted to validate such results. The criteria for classifying malignant SFT are still confusing and conventional. The signs of malignancy include tumour size > 10 cm, high mitotic activity, increased cellularity, and necrosis [33–35]. In addition, TERT promoter mutation [36] and BCOR mRNA overexpression [37] are correlated to the risk of malignancy in SFT.

However, histologically benign SFT can also have aggressive clinical features. The study of Gold, J. S. et al. have revealed that the rate of recurrence and metastasis of benign SFT were 6.7 and 5.3%, respectively [26]. Regardless of histology, a wide surgical excision is the most essential prognostic feature, both for benign and malignant tumours that behave in a clinically benign way as long as the tumour is completely resected with a clear surgical margin [4, 9]. Moreover, Sepideh Gholami et al. have indicated that tumour location is significantly associated with disease-specific mortality and individuals with tumours in the chest or abdominal/retroperitoneal cavity are at the highest risk [38].

In this report, we presented two tumour-related gene mutations according to the NGS analysis. PMS2 is one of the mismatch repair (MMR) genes [39], which causes genetic instability and is associated with tumour progression [40]. Deletion was believed to be the most frequent mutation of PMS2 [41, 42], which was previously referred to as hereditary nonpolyposis colorectal cancer [43] and other types of malignancy [44, 45]. However, beyond the deletion of PSM2, we did not observe any mutation in other MMR genes (MLH1, MSH2 and MSH6) in this case. In addition, the tumour was microsatellite stable. The correlation between this mutation and tumour invasiveness must be further assessed. ESR1 encodes oestrogen receptor α, which is a significant part of the nuclear hormone receptor family and is expressed in over two-thirds of patients with breast cancer [46]. In addition, it was considered an important tumour suppressor in several types of cancers, such as prostate cancer [47], osteosarcoma [48], non-small cell lung cancer [49] and hepatocellular carcinoma [50]. Interestingly, Hishida M. et al. have indicated that the decreased expression of ESR1 was correlated to pathological invasion of the intrahepatic portal vein (*p* = 0.0236) [50]. However, the L536P mutation (1607 T > C) was seldom reported [51], and its effects on gene expression and tumour invasion must be further validated.

As for the treatment, there is consensus showing that surgical resection is the most appropriate treatment for SFTs. However, the most intractable problems are recurrence and metastases after surgery [6]. In the study of Nicholas DeVito et al., the outcomes of systemic therapies, including chemotherapy and radiotherapy, were discouraging, which indicate that better systemic therapies are required, particularly in patients with metastatic/unresectable disease [6].

Metastases are usually blood borne and have been observed in sites, such as the liver [52], lung [53] and vertebra [54]. Due to the clinical rarity of intravascular SFT, the risk of metastasis has not been evaluated to date; hence, long-term clinical follow-up, which includes exploration and imaging, is required.

In summary, intravascular SFTs are extremely rare and generally present with luminal defects and changes in haemodynamics [55]. The imaging feature is lack of specificity, which can be missed or misdiagnosed [56]. In addition, several challenges are faced in terms of treatment. Hence, we presented this case involving gene variation as it can be used as reference in future studies.

## Conclusion

In this study, we first reported a case of solitary fibrous tumour located in the inferior vena cava. Moreover, NGS analysis was conducted, and two tumour-related mutations were found. We believe that our results might be useful in future studies of such disease.

## Data Availability

Not applicable.

## Abbreviations

CTV: Computed tomography venography
ESFT: Extrapleural Solitary Fibrous Tumor
FFPE: Formalin-fixed paraffin-embedded
IVC: Inferior vena cava
NGS: Next-generation sequencing
SFT: Solitary fibrous tumor
